# Serotonin syndrome induced by overdose of atomoxetine alone in a patient with attention‐deficit hyperactivity disorder: A case report

**DOI:** 10.1002/pcn5.41

**Published:** 2022-09-02

**Authors:** Fumika Sato, Akihito Suzuki, Keisuke Noto, Toshinori Shirata, Muneaki Kanno, Ryota Kobayashi, Koichi Otani

**Affiliations:** ^1^ Department of Psychiatry Yamagata University School of Medicine Yamagata Japan

**Keywords:** attention‐deficit hyperactivity disorder (ADHD), atomoxetine, overdose, serotonin syndrome

## Abstract

**Background:**

Serotonin syndrome is characterized by mental status changes, autonomic hyperactivity, and neuromuscular abnormalities. This syndrome results from various medications that engender serotonergic overactivity. Atomoxetine is a norepinephrine reuptake inhibitor used for the treatment of attention‐deficit hyperactivity disorder (ADHD). Two case reports have described serotonin syndrome induced by the combination of atomoxetine with venlafaxine or methylphenidate, but no report describes this syndrome induced by atomoxetine alone. This report describes serotonin syndrome induced solely by an overdose of atomoxetine in a patient with ADHD.

**Case Presentation:**

The patient in this case was a 21‐year‐old man who had been treated with atomoxetine for ADHD. He was transported to our hospital 1 h after intentional ingestion of 1200 mg of atomoxetine in a suicide attempt. On admission, he showed profuse diaphoresis, marked agitation, somnolence, slight fever, tachycardia, prolonged QT interval, myoclonus, tremor, and hyperreflexia. He was diagnosed as having serotonin syndrome and was treated with administration of activated charcoal and massive infusion. Three days later, his serotonin syndrome symptoms had disappeared completely.

**Conclusion:**

Findings in this case suggest that atomoxetine alone can cause serotonin syndrome presumably via its effects of serotonin reuptake inhibition. Clinicians should consider this syndrome induced by atomoxetine overdose.

## BACKGROUND

Serotonin syndrome is a potentially life‐threatening condition characterized by mental status changes (i.e., agitation and confusion), autonomic hyperactivity (i.e., fever, tachycardia, and diaphoresis), and neuromuscular abnormalities (i.e., myoclonus, tremor, and hyperreflexia).^1^, ^2^ This syndrome can be caused by various medications that engender serotonergic overactivity in the central and peripheral nervous systems.^1^, ^2^ Serotonin syndrome has been associated with widely various drug types, such as antidepressants, monoamine oxidase inhibitors, psychostimulants, and opioids.^1^

Atomoxetine is a selective norepinephrine reuptake inhibitor used to treat attention‐deficit hyperactivity disorder (ADHD).^3^ Two case reports have described serotonin syndrome associated with atomoxetine.^4^, ^5^ However, these cases were induced by the combination of atomoxetine with venlafaxine^5^ or methylphenidate,^4^ leading to the possibility that drugs other than atomoxetine might have contributed to this syndrome. This report describes serotonin syndrome induced solely by overdose of atomoxetine in a patient with ADHD.

## CASE PRESENTATION

The patient in this case was a 21‐year‐old man who had been treated with atomoxetine 80 mg/day at a local psychiatric hospital because of ADHD. No other medication had been administered. He reported that he had never used illegal drugs, and that he had rarely consumed alcohol. He provided written informed consent for presentation of his clinical course. This report has been approved by the Ethics Review Committee of Yamagata University Faculty of Medicine.

The patient was transported to the emergency unit of our hospital, 1 h after his intentional ingestion of 1200 mg of atomoxetine in a suicide attempt. On admission, he showed profuse diaphoresis, marked agitation, and somnolence. His body temperature was 37.4°C, blood pressure 137/74 mmHg, heart rate 135 beats/min, and corrected QT interval 0.604 s. Neurological examinations revealed severe myoclonus, tremor, and hyperreflexia of the four limbs. The laboratory blood test results were within normal levels except for increased levels of white blood cells (23,240/μl) and blood sugar (239 mg/ml). He had metabolic acidosis (pH = 6.880, PaCO_2_ = 55.0 mmHg, and HCO_3_^‐^ = 10.1 nmol/L). Chest X‐ray showed no abnormality. He was diagnosed as having serotonin syndrome according to the Sternbach criteria.^2^ Administration of activated charcoal, massive infusion, and conservative treatment were undertaken. Three days later, serotonin syndrome symptoms had disappeared completely. Blood test results were within normal limits. Seven days later, he was discharged.

## DISCUSSION

Serotonin syndrome is caused by serotonergic drugs, which can induce increased serotonin release, serotonin reuptake inhibition, serotonin metabolism inhibition, serotonin‐1a receptors activation, and serotonin‐2a receptors blockade.^1^ Earlier case reports have described serotonin syndrome associated with atomoxetine, venlafaxine,^5^ and methylphenidate^4^ co‐administered with atomoxetine. Venlafaxine and methylphenidate have the respective effects of serotonin reuptake inhibition^6^ and serotonin‐1a receptor activation.^7^ Therefore, the possibility cannot be excluded that venlafaxine or methylphenidate other than atomoxetine contributed to serotonin syndrome in these earlier cases. The case reported herein is the first describing that atomoxetine alone induced serotonin syndrome.

Atomoxetine is a selective norepinephrine reuptake inhibitor.^3^ However, an *in vitro* study using radioligand binding assays for human monoamine transporters showed that atomoxetine binds not only to the norepinephrine transporter, but also to the serotonin transporter to a lesser degree.^6^ A study using positron emission tomography in monkeys also showed that atomoxetine at clinically relevant doses occupies both norepinephrine and serotonin transporters.^8^ Therefore, in the case presented herein, the effects of atomoxetine on the serotonin transporter might have been caused by serotonergic overactivity, developing thereafter into serotonin syndrome.

Earlier reports have suggested that atomoxetine overdose at up to 1200 mg results in less severe toxicity and duration of the symptoms after overdose resolves in less than 30 h.^9^, ^10^ The patient in our case displayed severe serotonin syndrome at a dose of atomoxetine of 1200 mg. Moreover, his symptoms continued for 72 h after the overdose. These discrepancies suggest that our patient had individual predisposition to atomoxetine toxicity. Atomoxetine has been shown to be a substrate of cytochrome P450 (CYP) 2D6 and 2C19^3^; genetic variants of CYP2D6^11^ and 2C19^12^ are associated with impaired metabolism of atomoxetine. Therefore, it is possible that the patient in this case had genetic variants of CYP2D6 and/or 2C19. His genetically determined predisposition contributed to serotonin syndrome.

## CONCLUSION

In conclusion, results from this case suggest that atomoxetine can cause serotonin syndrome, presumably via its effects of serotonin reuptake inhibition. Clinicians should consider this syndrome induced by atomoxetine overdose.

## AUTHOR CONTRIBUTIONS

Fumika Sato, Akihito Suzuki, and Keisuke Noto treated the patient and drafted the manuscript. Toshinori Shirata, Muneaki Kanno, Ryota Kobayashi, and Koichi Otani critically reviewed the draft and revised it. All authors approved the final version of the manuscript.

## CONFLICT OF INTEREST

The authors declare no conflict of interest.

## ETHICS APPROVAL STATEMENT

Written informed consent for presentation of clinical course was given by the patient. This report has been approved by the Ethics Review Committee of Yamagata University Faculty of Medicine.

## Data Availability

All relevant data are included in the paper.
